# Editorial: Perspectives in Mammary Gland Development and Breast Cancer Research

**DOI:** 10.3389/fcell.2020.00719

**Published:** 2020-08-25

**Authors:** Vida Vafaizadeh, Emilia Peuhu, Alexandra Van Keymeulen, Zuzana Koledova

**Affiliations:** ^1^European Molecular Biology Laboratory (EMBL), Genome Biology Unit, Heidelberg, Germany; ^2^Institute of Biomedicine and Cancer Research Laboratory FICAN West, University of Turku, Turku, Finland; ^3^Turku Bioscience Centre, University of Turku and Åbo Akademi University, Turku, Finland; ^4^Laboratory of Stem Cells and Cancer, Faculty of Medicine, Université Libre de Bruxelles (ULB), Bruxelles, Belgium; ^5^Department of Histology and Embryology, Faculty of Medicine, Masaryk University, Brno, Czechia

**Keywords:** breast cancer, mammary gland, development, imaging, microenviroment, organoid, signaling, omics

Annually, researchers from around the world, who work in the field of mammary gland biology and breast cancer and related areas, have a great opportunity to meet and discuss their work at a conference organized by the *European Network of Breast Development and Cancer* (ENBDC). These meetings, entitled *Annual ENBDC Workshop: Methods in mammary gland biology and breast cancer*, are largely methodologically oriented. They enable not only presentation of the latest scientific results, but also dissemination of cutting-edge approaches and forefront technologies that have facilitated these discoveries. The latest meeting took place on the 16th to 18th of May 2019 in Weggis, Switzerland, and presented exciting findings achieved using high resolution ‘omics approaches, genetic mouse models, organoids, and state-of-the-art imaging (Vafaizadeh et al., 2019). Here, we present a collection of articles based on or related to the topics of the ENBDC workshop.

Breast cancer is the most common cancer in women, annually diagnosed in more than 2.1 million women worldwide and more than 650,000 women worldwide die from this heterogeneous disease every year. To improve treatment strategies, deep understanding of breast cancer and metastasis is required. In their review, Parsons and Francavilla discuss how genomics, transcriptomics, proteomics, and metabolomics datasets, in combination with traditional breast cancer models, provide insights into breast cancer biology and enable discovery of novel therapeutic targets or biomarkers. They also emphasize the importance of transparent data sharing in data repositories to allow further meta-analysis and potential discoveries of previously unnoticed biomarkers or therapeutic targets. Waterhouse et al. further discuss the challenges of targeting driver oncogenes of triple negative breast cancer (TNBC). They suggest that identification of protein-protein interactions of TNBC oncogenes is needed to understand their functions in TNBC and to reveal novel therapeutic targets. They provide a nice overview of current and emerging agents for targeting TNBC oncogenes on cell surface, cytoplasm, and nucleus, including different genetic and epigenetic strategies for targeting transcription factors.

Three of the articles are focused on specific signaling pathways in breast cancer. van Schie and van Amerongen highlight the role of aberrant WNT-CTNNB1 signaling in human breast cancer and discuss three major gaps in this field: (i) Incomplete understanding of WNT signaling functions in normal human breast development and physiology, (ii) lack of knowledge of the extent and effect of (epi)genetic changes in the WNT pathway in different breast cancer subtypes, and (iii) lack of insight and biomarkers for selection of the correct subset of patients who might benefit from WNT pathway therapeutics. Fang et al. reviewed the roles of genes in the Fanconi Anemia pathway, which plays a central role in repairing DNA interstrand cross-links and includes the well-known DNA repair proteins BRCA1 and BRCA2. The authors describe promising strategies, like synthetic lethality, to target Fanconi Anemia pathway for breast cancer therapy. A new insight on how STRIP1, a component of the STRIPAK kinase and phosphatase complex, contributes to breast cancer regulation, is provided by Rodriguez-Cupello et al.. They observed increased viability in STRIP1-depleted breast cancer cells after chemotherapy treatment compared to control cells, and detected high induction of the CDK inhibitor p21 via MST3/4 kinases in STRIP1-depleted cells, which appeared to provide protection from treatment-induced DNA damage. These observations suggest that loss of STRIP1 can promote recurrent disease after treatment with sub-optimal doses of chemotherapy.

Microenvironment plays an important role in tissue homeostasis, cancer progression, and metastasis (Bissell and Hines, 2011). Cancer-associated stroma (CAS) is composed of different cellular and extracellular components. Understanding transcriptional reprogramming of CAS is crucial for efficient targeting of tumor progression. Spontaneous canine simple mammary tumors (CMTs) are a useful model of human breast cancer to study the reprograming of CAS in malignant carcinomas compared to benign adenomas. In her article, Markkanen provides evidence for molecular homologies in stromal tissues between canine and human mammary tumors.

Disseminated breast cancer cells can survive for a long period in a foreign environment without developing into overt metastasis (Park and Nam, 2020). Detection and eradication of these cells is imperative to avoid cancer relapse. Montagner and Sahai have assembled a very useful overview about the current *in vitro* models to study breast cancer dormancy. They highlight the challenges of development and validation of the models, discuss the role of different dormant niche components, and present the models developed for metastatic breast cancer dormancy in different tissues, such as lung or bone.

The immune checkpoint blockade (ICB) therapy (Pardoll, 2012) is one of the promising approaches in personalized breast cancer therapy. Vafaizadeh and Barekati summarized recent studies on immuno-oncology biomarkers, which are crucial for selection of responsive cancer patients to ICB, such as anti-PD1 and anti-PD-L1 antibodies, to achieve clinical benefit.

To fully understand the defects leading to breast cancer, it is essential to decipher the mechanisms that regulate normal mammary epithelial morphogenesis and homeostasis. Mammary gland consists of a branched network of epithelial tubes embedded in a complex stroma. The three-dimensional (3D) epithelial architecture is critical for proper mammary function. Therefore, to study mammary morphogenesis and dynamics, 3D cell cultures are essential (Weigelt et al., 2014; Koledova, 2017). To this end, Sumbal et al. developed a new *ex vivo* model of mammary lactation and involution using primary mouse organoids. This model can be applied to study mechanisms of physiological mammary gland lactation and involution as well as pregnancy-associated breast cancer. Budkova et al. investigated regulation of epithelial-to-mesenchymal transition (EMT), a developmental process that is often hijacked by cancer cells. They found that maternally expressed non-coding RNAs of the *DLK1-DIO3* locus are markers of EMT and that MEG3 is a novel regulator of EMT/MET in breast tissue.

Mammary stroma provides instructive signals for mammary gland morphogenesis and homeostasis (Wiseman and Werb, 2002). Macrophages are one of the cellular components of the stroma, implicated in regulation of all stages of mammary gland development (Schwertfeger et al., 2006). Using optical tissue clearing and 3D imaging of mammary tissue obtained from *Csf1r-EGFP* mice, Stewart et al. revealed stage-specific differences in macrophage abundance, localization, morphology, and association with epithelial cells. Their article provides important insights into dynamics of macrophage distribution during mammary gland development and demonstrates the need for high-resolution, multidimensional imaging approaches to study the highly dynamic mammary gland morphogenesis. The current and state-of-the-art imaging approaches, instrumental to shedding light on mammary gland ductal development, lactation, as well as tumor invasion and metastasis, are review by Lloyd-Lewis. She discusses advantages of several fluorescence light-based microscopy platforms and considers specific technical requirements for intravital imaging as well as fixed tissue processing, including clearing.

In summary, this Research Topic includes both original research articles as well as review articles and reflects the wide range of current research in the mammary gland biology and breast cancer fields. We hope that they will be of interest to a broad scientific readership.

## Author Contributions

All authors listed have made a substantial, direct and intellectual contribution to the work, and approved it for publication.

## Conflict of Interest

The authors declare that the research was conducted in the absence of any commercial or financial relationships that could be construed as a potential conflict of interest.
